# Pilot Study to Evaluate Hearing Aid Service Delivery Model and Measure Benefit Using Self-Report Outcome Measures Using Community Hearing Workers in a Developing Country

**DOI:** 10.1155/2013/973401

**Published:** 2013-02-06

**Authors:** Lingamdenne Paul Emerson, Anand Job, Vinod Abraham

**Affiliations:** ^1^Unit I, Department of ENT, Christian Medical College, Vellore 632001, India; ^2^Department of CHAD, Christian Medical College, Vellore 632001, India

## Abstract

Hearing loss is a major handicap in developing countries with paucity of trained audiologists and limited resources. In this pilot study trained community health workers were used to provide comprehensive hearing aid services in the community. One hundred and eleven patients were fitted with semi-digital hearing aid and were evaluated over a period of six months. They were assessed using self-report outcome measure APHAB. Results show that trained CHWs are effective in detecting disabling hearing loss and in providing HAs. APHAB can identify and pick up significant improvements in communication in daily activities and provides a realistic expectation of the benefits of a hearing aid. The model of using trained CHWs to provide rehabilitative services in audiology along with self-report outcome measures can be replicated in other developing countries.

## 1. Introduction

It is well recognized that hearing loss is the most common global disability [1]. As reported by Davis [2] (British MRC Institute of Hearing Research) the numbers with hearing impairment in developing countries is estimated to reach 500 million by the year 2015. In India, hearing loss is the third most common disability [3] and the prevalence of moderate to severe hearing loss is reported to be 6.3 percent by WHO [4]. The majority of India's population lives in rural areas with poor facilities and accesses for evaluation of hearing and treatment being seldom available. Also, due to the paucity of trained audiologists, [5] the burden of hearing handicap is magnified. The primary goal of the study was to determine the degree and nature of benefit that could be expected in the population by using trained community workers to fit semidigital hearing aids which can be programmed easily by trained workers and quantifying benefit using APHAB which was translated in to the local language.

Deafness is an expensive handicap, leading to loss of work and active participation in social activities of the community [6].

The measurement of outcomes in rehabilitative audiology has received more attention because of the need to demonstrate efficacy of treatment for consumers, carry out cost-benefit analyses. Traditionally, the outcomes of hearing aid intervention have been demonstrated using objective measures such as the functional gain [7] speech recognition [8] testing, and real ear responses [9]. An alternative to the use of objective hearing aid benefit measurements is the use of self-report methodology. In the 1980s and 1990s several other subjective measurement instruments were developed to assess hearing aid benefit [10, 11].

One of the most common subjective benefit measures in use today is the Abbreviated Profile of Hearing Aid Benefit (APHAB) developed by Cox and Alexander [12].

APHAB is a questionnaire which has 24 predetermined questions regarding various situations of hearing aid usage. It also gives the patient a realistic estimation of the use of a hearing aid. It is scored before and after fitting hearing aids (Table 3). The difference in scores with and without the use of hearing aid(s) is considered the measure of benefit. There are four categories in which benefit is calculated: ease of communication (EC), listening in background noise (BN), listening in reverberant conditions (RV), and aversiveness of sounds (AV).

In a large developing country like India with significant rural population, the use of community health workers to identify hearing loss and provide hearing aids and associated services and evaluate the use and satisfaction of HAs by self-report measure APHAB and its stability was studied over a period of six months.

## 2. Methodology

### 2.1. Selecting the Health Workers

Community Hearing Workers (CHWs) were selected with educational qualification of graduate with preference for science subjects. They were given a six week training program on basic hearing health care, which included performing Pure tone audiometry (PTA) using a portable audiometer, impression taking, and ear mould making, performing hearing aid trial and hearing aid fitting, maintenance of hearing aids, minor repairs of hearing aids, and maintenance of ear moulds. They were also taught to counsel patients regarding hearing aid usage.

They were also trained to administer APHAB, translated into the local language (Tamil) and validated by the audiologist.

### 2.2. Recruiting of Patients

Camps were conducted with the help of the local government organization and nongovernmental agencies (NGOs), using local propaganda machine which included television broad casting and advertising in the regional language papers. Patients were screened by an ENT specialist and hearing assessment was done in the field using ARPHI-500 MKI dual channel portable audiometers programmed to ANSI standards in a quiet room. A three frequency (0.5 kHz, 1 kHz, 2 kHz) average was taken and hearing loss was classified as moderate, moderately severe, severe, and profound hearing loss. The PTA was later counter checked by the audiologist at the base hospital.

 Inclusion criteria: age: 14–70 years.
 Hearing loss—bilateral moderate to severe sensorineural hearing loss (41–90 dB).
 Exclusion criteria:
  1. Hearing loss which can be surgically corrected.
  2. Children below 14 years and adults above 70 years.
  3. Mild hearing (<40 dB) loss and profound (>90 dB) hearing loss.

## 3. APHAB

The APHAB is a hearing aid outcome measure that reflects the impact of hearing loss and benefit of hearing aid in daily communication.

The APHAB comprises 24 items that are scored in 4 subscales. The subscales are as follows.

1. Ease of communication (EC): the strain of communicating under relatively favorable conditions.
2. Reverberation (RV): communication in reverberant rooms such as class rooms.
3. Background noise (BN): communication in settings with high background noise levels.
4. Aversiveness (AV): the unpleasantness of environmental sounds.

There are 24 items; each item contributes to only one subscale and there are six items for each subscale distributed randomly with in the inventory. Each item is answered for “without my hearing aid” and “with my hearing aid” so that each subscale produces a score for unaided listening and a score for aided listening. In addition, the difference between these two scores can be obtained to give a score for benefit. The complete APHAB generates 12 scores, three for each of four subscales.

The difference in the rating between the initial and the final visit is scored as the amount of benefit in each of four general categories.

The first three subscales, EC, RV, and BN, are known as the “speech communication” subscales. These subscale benefit scores are reported in the form of a positive percentage (i.e., 10%, 20%, 30%). The fourth subscale, aversiveness (AV), quantifies an individual's negative reaction to aversive environmental sounds. This subscale is reported in a negative percentage (i.e., −10%, −20%, −30%).

Hearing aids (Siemens 213) were dispensed based on the formula

(1) NH=3 khz−500 hz,MPO≥60  decibel=0.3×3  FA+89<60  decibel=0.53×3  FA+75.

## 4. Procedure

After identifying suitable individuals, a detailed session of counseling was done and APHAB questionnaire was filled up prior to HA fitting by the CHWs. Hearing assessment was done and ear mould impression was taken for selected patients. In the second visit, a semidigital, trimmer programmable hearing aid, Siemens Phoenix 213, which is a mini-BTE, suitable for moderate to severe hearing loss, was fitted for all the patients. The HAs were programmed to NAL (National Acoustic Laboratory) prescription standards [13]. Adjusting the HA for optimal use and its maintenance was taught. APHAB followup was done for 2 weeks, one month, three months, and six months. Modern telecommunication facilities were used and mobile phone was used by the patients to communicate with the health workers and minor problems of moulds and hearing aid fitting and trimmer adjustments were done in the field. The hearing health care service was provided free of health care costs to the patients at their doorstep by the CHWs.

## 5. Results

This study was done to evaluate a model of using trained Community hearing workers to provide rehabilitative audiological services in the community and to asses the use of Hearing Aids and their benefit in the community by using self reported benefit questionnaire APHAB translated in to local language.

**Figure 1:**
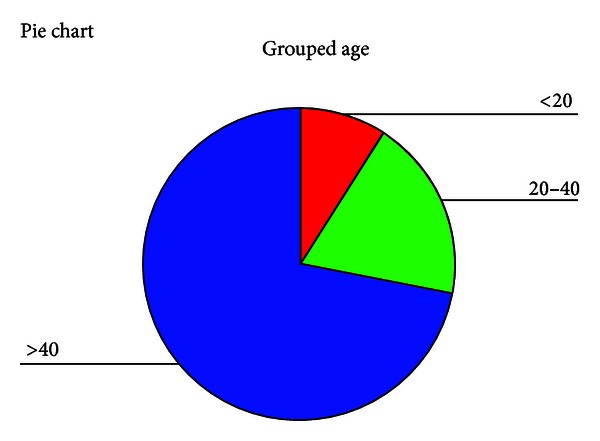
Distribution of age.

A total of 111 patients with 48 females and 63 males, having moderately severe to severe ((41–90 dB) sensori-neural hearing loss (Table 1) were rehabilitated with hearing aids from March 2009 to September 2009. All were first time users with no prior hearing aid experience. 57% were males and 72.9% were above the age of 40 years (Table 2) (Figure 1). The whole process from the identification of patients to fitting hearing aids and followup over six months was done in the community.

105 (94.5%) patients came for followup to six months. Six (5.4%) patients did not come for regular followup.

Of the six patients (5.4%) who failed to come for regular followup, one was later found to have auditory neuropathy. Two patients complained of excessive noise, and two did not have benefit from the HA.

88 patients (80%) were using their hearing aid regularly for more than 4 hours, whereas 20% were using it for less than 4 hours, due to their profession, which involved working as laborers, where sweating of face and dirt on hands were constraints for continuous usage.

It took approximately 25 minutes to administer APHAB.

 The questionnaire when administered before HA fitting (unaided), 108 (97%) answered all the questions with respect to ease of communication (EC), background noise (BN), and aversiveness (AV). However, 14% did not respond to all the questions which assess reverberation (RV). There was no significant difference in the response pattern with respect to gender in both unaided and aided scores. It took time to administer as patients were semiliterate/illiterate in addition to being hearing challenged.

**Figure 2:**
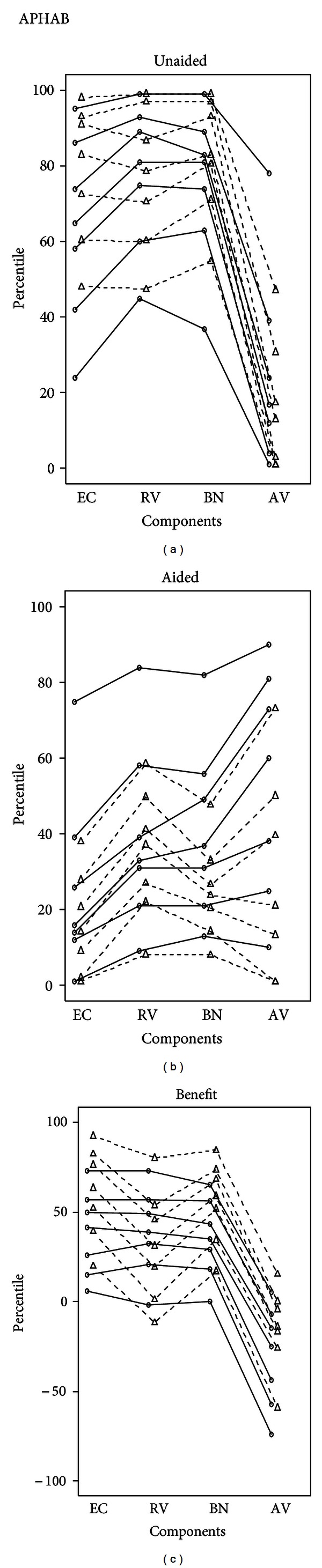
On comparing the percentiles of the present study with those of normative data [12].

It was found that clients experienced more problems in personal communication (EC) even with little or no background noise with greatest difficulty faced when talking with family and friends. Communication in crowded places /shop (BN) experienced greatest benefit with most people being able to participate in community actively. They were able to participate in the social activities of the community (RV) and also enjoy recreational activities (movie) and also participate in religious activities (Figure 2).

Over a period of six months the mean scores with respect to EC were almost similar to that assessed at the end of two weeks. Patients experienced fewer problems while communicating in background noise, whereas the mean scores with respect to communication in reverberant surroundings were more when compared with two weeks post fitting scores. Scores for Aversiveness increased in comparison to immediate post fitting scores.

Clients without hearing aid reported more than 35th percentile problems with respect to EC (85%), BN (79.2), RV (73%). 68.4% scored less than 50th percentile with respect to aversive sounds in the environment (AV).

**Figure 3:**
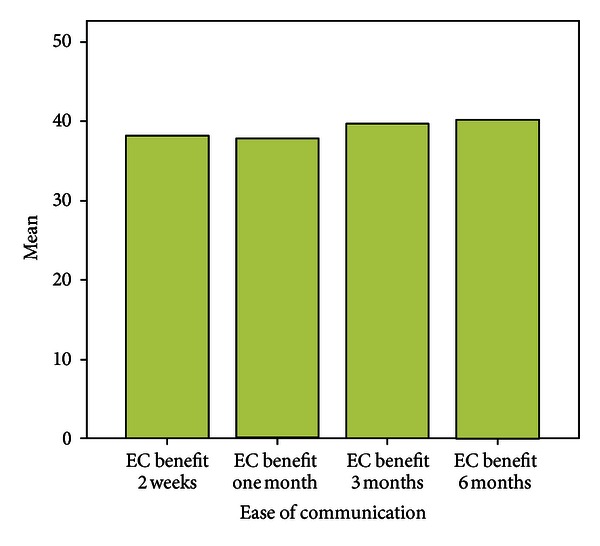
Benefit over six months.

**Figure 4:**
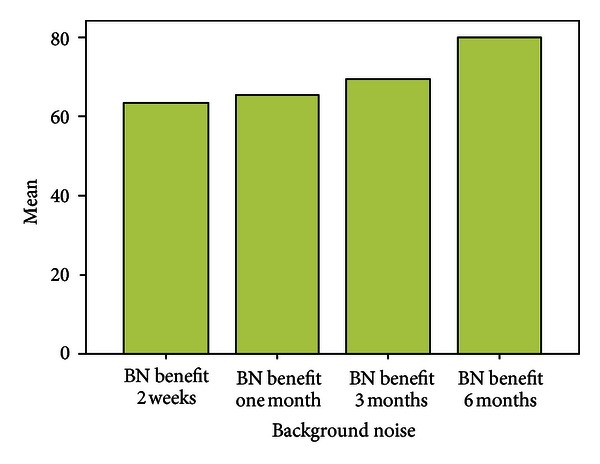
Benefit over six months.

**Figure 5:**
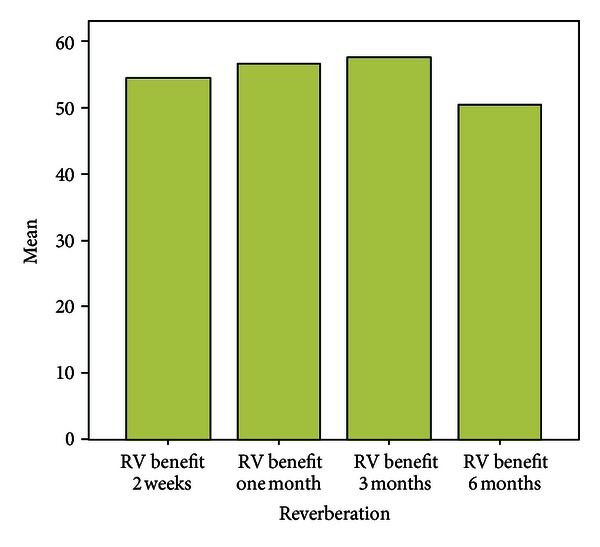
Benefit over six months.

**Figure 6:**
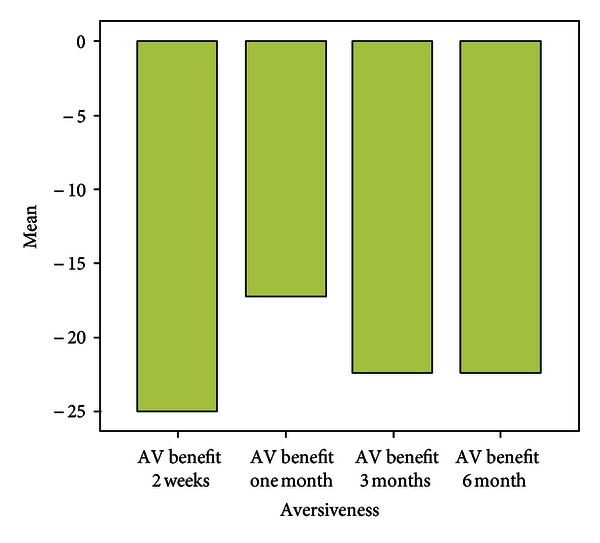
Benefit over six months.

Problems in the speech communication subscales (EC, BN, and RV) showed a significant decrease after using the hearing aid (Figures 3, 4, 5, and 6).

**Figure 7:**
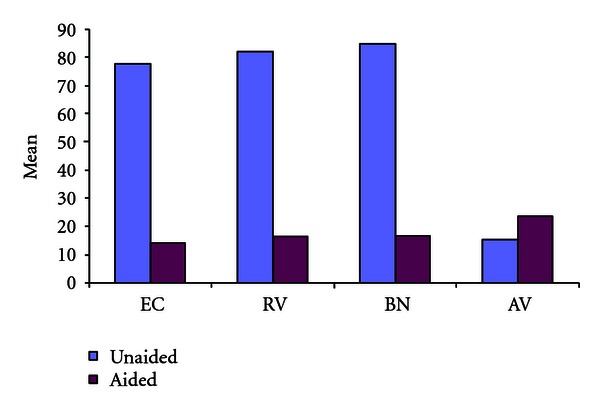
Mean scores.

There was significant benefit >25 in all the three speech subscales (Figure 7).

## 6. Discussion

The main purpose of this study was using trained community workers to provide hearing aids in the community and to assess the self-report measure APHAB in the community and its stability over a period of six months.

It was found that most hearing aid users were above the age of 40, which was similar to the other studies. Hearing aids were fitted for patients with moderate to severe hearing loss which had a high acceptance level (98%).

Most of the recruited patients were from cluster of surrounding villages. Thus during followup patients were called together in the village and they could discuss the problems they faced and how to resolve them. The issues regarding usage of hearing aid and the stigma of not being able to hear were sorted out among themselves. A feeling of community helped them to overcome the shyness of using a hearing aid and thus it led to increased acceptance and the despondency of being a deaf person was overcome which led many other people with similar problem to seek help for their problem. In APHAB, in contrast to the report by Hojan et al., there was no significant difference in responses between male and female HA users [14].

There was a significant benefit in all the three communication subscales. The missing responses to BN and AV scales could be explained from the fact that the mentioned situations were not encountered immediately after hearing aid fitting and may have been due to rural living, which did not expose them to situations where background noise and aversive sounds were present.

The AV subscale score is typically higher at post-fitting. This likely reflects the increased audibility of certain environmental sounds that even individuals with normal hearing may find aversive. The small, statistically nonsignificant change in the AV scores indicates that the hearing aid provided acceptable listening comfort and sound quality for most of the participants.

There was correlation between the aided condition and PTA thresholds as reported by Cox et al. [15] which confirms that while fitting hearing aids in the community, clients with moderately severe and severe hearing loss suffer more problems which can be alleviated with provision of hearing aids suited for that range that is, the hearing aid has to be compatible or provide significant gain with respect to speech communication subscales (EC, RV, BN).

APHAB picked up significant improvements in communication especially in reverberant rooms and in settings with high background noise levels.

The limitation of this study was the lack of responses to questions, as in a rural community the provision of modern amenities is lacking when compared to an urban setting.

## 7. Conclusion

APHAB picked up significant improvements in communication especially in reverberant rooms and in settings with high background noise levels. Though the questionnaire is long the questions take into consideration most of the situations the patients face in their daily life. However in a rural community with lack of basic amenities some of the questions may not be assessed for a considerable time period till the patient comes into contact with such situations.

However it is the most comprehensive questionnaire and gives the patient a realistic expectation from a hearing aid. It can also be used at a later time when the patient upgrades his hearing aid and thus the benefits can be compared.

## Figures and Tables

**Table 1:** Gender versus degree of hearing loss.

	Moderately severe	Severe	Profound	
Sex				
F	16	30	2	48
M	20	42	1	63

Total	36	72	3	111

**Table 2:** Age versus degree of hearing loss.

	Moderately severe	Severe	Profound	
Age_1				
14–25	6	11	0	17
26–60	19	32	2	53
61–70	11	29	1	41

Total	36	72	3	111

**Table 3:** Scores for each question.

	Unaided		Aided		Benefit	
	Mean	Std. deviation	Mean	Std. deviation	Mean	Std. deviation
BN 1	89.39	14.786	20.85	19.128	67.83	22.814
BN 6	84.57	20.355	15.03	17.070	69.43	29.853
BN 7	83.17	23.400	12.86	19.386	70.31	32.893
BN 16	90.07	16.572	27.18	24.825	61.41	27.376
BN 19	93.25	11.208	17.42	16.498	74.48	19.199
BN 24	73.23	35.959	19.27	23.300	53.72	44.147

EC 4	81.94	18.703	16.84	24.216	65.10	34.362
EC 10	74.53	32.162	11.28	20.874	66.14	38.632
EC 12	77.95	27.126	10.54	16.133	68.05	28.147
EC 14	82.01	25.096	21.32	23.844	59.28	32.167
EC 15	72.39	32.067	18.99	27.937	51.55	49.414
EC 23	79.09	24.532	12.50	16.149	66.63	28.935

RV 2	82.32	29.281	27.25	23.335	56.02	40.909
RV 5	83.96	21.608	15.60	18.732	67.64	32.068
RV 9	88.99	16.719	15.09	14.781	73.63	21.607
RV 11	93.59	11.424	17.79	19.929	72.61	26.129
RV 18	70.84	35.135	26.79	31.958	42.17	61.785
RV 21	89.36	17.287	23.44	21.319	64.88	28.673

AV 3	15.89	31.728	26.80	31.202	−11.64	45.396
AV 8	22.76	35.284	28.50	32.159	−5.79	46.441
AV 13	18.06	23.689	23.59	36.102	−4.38	35.431
AV 17	15.85	29.795	34.36	34.442	−17.82	49.513
AV 20	9.65	22.504	32.22	34.550	−27.09	36.752
AV 22	5.73	15.528	27.78	31.694	−22.46	31.084

## Abbreviations

APHAB: Abbreviated Profile of Hearing Aid Benefit
PTA: Pure tone audiometry
HA: Hearing aid
CHW: Community hearing worker
MPO: Maximum Power output
FA: Frequency average.
